# Overall survival analysis in patients with metastatic breast cancer and liver or lung metastases treated with eribulin, gemcitabine, or capecitabine

**DOI:** 10.1007/s10549-020-05867-0

**Published:** 2020-08-17

**Authors:** Shayma Kazmi, Debanjana Chatterjee, Dheeraj Raju, Rob Hauser, Peter A. Kaufman

**Affiliations:** 1 Cancer Treatment Centers of America, Philadelphia, PA USA; 2 US Health Economics Outcomes Research and Real World Evidence, Eisai Inc., Woodcliff Lake, NJ USA; 3 Cancer Treatment Centers of America Global, Inc., Boca Raton, FL USA; 4 Larner College of Medicine, Division of Hematology/Oncology, University of Vermont Cancer Center, Burlington, VT USA

**Keywords:** Metastatic breast cancer, Visceral metastases, Overall survival, Eribulin, Real-world evidence

## Abstract

**Purpose:**

The purpose of this study was to estimate the overall survival (OS) in real-world clinical practice in patients with metastatic breast cancer (MBC) and visceral metastases (liver or lung) treated in the third-line setting with eribulin, gemcitabine or capecitabine overall and in the major clinical categories of MBC (TNBC, HR+/HER2−, and HER2+).

**Methods:**

A retrospective, observational study was conducted with de-identified patient electronic health records from the Cancer Treatment Centers of America (CTCA). Patients with a diagnosis of metastatic breast with lung or liver metastases, and treated with eribulin, gemcitabine, or capecitabine as third-line therapy were included in the analysis. Landmark survival was calculated as percentage of patients alive at 6, 12, 24, and 36 months. Overall survival was compared between treatment arms within TNBC and HR+/HER2− using log-rank analysis. Cox regression analyses was performed to estimate hazard ratios for comparison of treatments within TNBC and HR+/HER2− subtype.

**Results:**

443 patients with liver or lung metastases received third-line therapy with eribulin (*n* = 229), gemcitabine (*n* = 134), or capecitabine (*n* = 80). Eribulin patients had a higher percentage of patients alive at all landmark timepoints vs. gemcitabine, and a higher percentage of patients alive until 36 months vs. capecitabine. Median survival times showed that overall, and within the TNBC and HR+/HER2− subtype, patients receiving eribulin had a numerically higher median overall survival.

**Conclusions:**

This real-world evidence study is consistent with randomized clinical trial data and demonstrates consistency of eribulin effectiveness in MBC patients with lung or liver metastases overall and in TNBC and HR+/HER2− disease.

## Background

An estimated 268,600 cases of invasive breast cancer were diagnosed in 2019, and approximately 6% of these were stage 4, or de novo metastatic breast cancer (MBC) [1]. For patients diagnosed with earlier stages of breast cancer, approximately 30% will eventually develop recurrent advanced or metastatic disease [2]. Although survival benefits have been observed in patients with MBC over the past several decades [3], 5 year survival rates remain approximately 27% [1], and the disease remains incurable [3].

Prognosis among patients with MBC varies based on hormone receptor (HR) and human epidermal growth factor receptor 2 (HER2) status. Patients with triple-negative breast cancer (TNBC) have been shown to have a substantially shortened median survival when compared to patients with hormone receptor positive (HR+, HER2−) and HER2+ breast cancer [4, 5]. Metastatic patterns of breast cancer have also been shown to vary by hormone receptor status. TNBC patients have an increased incidence of visceral and cerebral distant metastases [4]. Furthermore metastatic breast cancer patients with visceral metastases to the liver and/or lung have a particularly poor prognosis, extent of which could differ by breast cancer subtype [6]. As per the National Comprehensive Cancer Network (NCCN) guidelines version 2.2020, women with HR- MBC tumors not localized to bone or soft tissue only, that are associated with symptomatic visceral metastasis or those that have HR+ MBC that is refractory to endocrine therapy, should receive chemotherapy [7].

There are several chemotherapeutic agents available that have been shown to be effective either as single agents or in combination for the treatment of MBC [7]. Therapy selection should be based on individual patient factors (e.g. previous chemotherapy exposure and response, comorbid conditions, patient preference, etc.). Additionally, route of administration could also be a consideration in therapy selection. Intravenous (IV) agents may help to avoid dosing mistakes (i.e. patients forgetting treatment breaks or taking incorrect doses), adherence issues, handling or storage issues, and adverse event management [8]. Eribulin, gemcitabine, and capecitabine are three agents used in the MBC setting that offer different routes of administration. Eribulin, an IV microtubule inhibitor, was first approved in the U.S. in 2010 for the treatment of patients with metastatic breast cancer who have previously received at least two chemotherapeutic regimens for the treatment of metastatic disease. Prior therapy should have included an anthracycline and a taxane in either the adjuvant or metastatic setting [9]. Gemcitabine, an IV pyrimidine antimetabolite, was approved in the United States in 2004 in combination with paclitaxel, and is indicated for the first-line treatment of patients with metastatic breast cancer after failure of prior anthracycline-containing adjuvant chemotherapy, unless anthracyclines were clinically contraindicated [10]. Gemcitabine is recommended as a preferred single agent in HER2-ve metastatic breast cancer as per NCCN guidelines [7]. Capecitabine is an oral nucleoside metabolic inhibitor approved in 1998 in combination with docetaxel after failure of prior anthracycline containing therapy and as monotherapy in patients resistant to both paclitaxel and an anthracycline-containing regimen [11]. In addition, capecitabine was approved in 2007 for use with lapatinib, for patients with HER2+ MBC and who have received prior therapy including an anthracycline, a taxane, and trastuzumab [11, 12].

A pooled analysis from two phase 3 trials of patients with MBC treated with eribulin versus control chemotherapy (capecitabine or treatment of physician’s choice (TPC) after 0–5 prior chemotherapy regimens, including an anthracycline and a taxane in the early or advanced setting) demonstrated improvement in overall survival (OS) favoring eribulin [13]. In the phase 3 trial comparing eribulin to capecitabine, a numerical trend favoring eribulin in OS was noted, that was not statistically significant [14]. While clinical trial data has thus overall shown a survival benefit in patients treated with eribulin, there is sparse information available (particularly in the U.S.) to demonstrate this benefit in a real-world setting [3]. The purpose of this study was to estimate the OS in real-world settings in patients with MBC and visceral metastases (liver or lung) who were treated specifically in the third-line setting with eribulin, gemcitabine or capecitabine in the United States overall and further analyzed by receptor status (TNBC, HR+/HER2−, and HER2+).

## Methods

### Study design and data source

A retrospective, observational study was conducted with de-identified patient electronic health records (EHRs) from the Cancer Treatment Centers of America (CTCA). The CTCA consists of a network of five comprehensive cancer care and research centers throughout the U.S. The CTCA focuses on integrated, patient-centered comprehensive cancer care. Data used in this study included information on patients from over 39 community oncologists across the U.S. and covered the time period from January 1, 2012 through October 13, 2018. The study was approved by the Institutional Review Board.

### Study population and determination of line of therapy

Patients who met the following inclusion criteria were included in the analysis:

- breast cancer diagnosis as defined by an ICD-9 or ICD-10 code
- confirmed stage 4 disease (as per the American Joint Committee on Cancer diagnostic criteria) or recurrent/metastatic disease
- lung or liver site of metastases as confirmed by radiographic records
- treated with eribulin, gemcitabine, or capecitabine as third-line therapy

The first prescription or administration date of eligible anticancer therapy was the index date. All anticancer therapy prescribed/administered/documented within the first 30 days of the start date of a line of therapy (i.e. index date + 29 days) was considered to be part of that line of therapy. The end date of therapy was defined as the earliest of any of the following: physician documentation that a treatment had been discontinued, addition or substitution of a new agent after the initial agent, a treatment gap of ≥ 60 days after the runout date of all agents in the line of therapy, death, or end of the study period. Instances where a new agent was added or substituted, the end date was defined as the day prior to the start of the new agent. Discontinuation of one agent from a multidrug regimen did not qualify as ending that line of therapy. In instances where a treatment gap of ≥ 60 days was noted, the line of therapy end date was the last date prior to the gap. The duration of treatment was calculated as the time period between the start and the end of treatment. Patients were grouped into treatment cohorts for the analysis based on the third-line therapy received and were also stratified by hormone receptor subtype.

### Statistical analyses

Patient demographics and clinical characteristics were reported by treatment. Overall Survival (OS) was the primary outcome measured in the analysis. Landmark survival percentages at 6, 12, 24, and 36 months and median OS (95% CI) were reported by treatment and stratified by hormone receptor status. Kaplan–Meier analyses were conducted by treatment arm within the TNBC and HR+/HER2− hormone receptor subgroups. Overall survival for patients treated with eribulin were compared with gemcitabine and capecitabine respectively using Kaplan–Meier curves with log-rank analysis. Patients alive at data cut-off were censored using the last assessment date. Median overall survival was estimated for each treatment arm overall and by hormone receptor status. Cox regression analysis was implemented to estimate hazard ratios for comparison of treatments within each hormone receptor subtype. All analyses were conducted using SAS, v9.4.

## Results

There were 1828 patients initially identified with MBC. Of these, 1471 had visceral metastases (liver or lung), and 443 received third-line therapy with eribulin (*n* = 229), gemcitabine (*n* = 134), or capecitabine (*n* = 80) (Table 1). In the cohort of patients treated with eribulin, the majority were Caucasian (52%) and had ECOG status of 0 or 1 (69%) with an average age of 55 years. The distribution of hormone receptor subtypes in the eribulin group was TNBC (29%), HR+/HER2− (62%), and HER2+ (9%). The three treatment cohorts were similar in age and ethnicity, though a higher proportion of ECOG 0 and HER2+ subtype in the capecitabine cohort were observed (Table1).

**Table 1:** Demographics and clinical characteristics by treatment

	Eribulin (*n* = 229)	Gemcitabine (*n* = 134)	Capecitabine (*n* = 80)
Age (years), mean (SD)	55.0 (8.63)	55.9 (9.49)	55.5 (9.7)
Race, *n* (%)			
Caucasian	120 (52.40)	69 (51.49)	42 (52.50)
African American	84 (36.68)	59 (44.03)	29 (36.25)
Other	25 (10.92)	6 (4.48)	9 (11.25)
ECOG performance status, *n* (%)			
0	39 (17.03)	15 (11.19)	23 (28.75)
1	119 (51.97)	63 (47.01)	34 (42.50)
2	53 (23.14)	43 (32.09)	9 (11.25)
3	12 (5.24)	9 (6.72)	5 (6.25)
4	1 (0.44)	0	1 (1.25)
Unknown	5 (2.18)	4 (2.99)	8 (10.00)
Hormone receptor and HER2 subtype^a^, *n* (%)			
TNBC	66 (28.82)	36 (26.87)	20 (25.00)
HR+/HER2−^b^	142 (62.0)	77 (57.5)	41 (51.3)
HER2+ ^c^	21 (9.17)	21 (15.67)	19 (23.75)

*ECOG* Eastern Cooperative Oncology Group, *ER* estrogen receptor, *PR* progesterone receptor, *HER2* human epidermal growth factor receptor 2, *HR* hormone receptor, *SD* standard deviation, *TNBC* triple negative breast cancer and is defined as ER−, PR− and HER2−

^a^These groups are designed to be mutually exclusive

^b^HR+/HER2− includes ER+ and/or PR+ and HER2−

^c^HER2+ includes ER+ and/or PR+ and HER2+

Landmark survival at 6, 12, 24 and 36 months for all patients treated with eribulin was 65%, 38%, 14%, and 7%, respectively. Corresponding landmark survival for each time point by hormone receptor subtype for patients treated with eribulin were as follows: for TNBC: 49%, 35%, 20%, and 8%, respectively, for HR+/HER2−: 72%, 42%, 11%, and 7%, respectively, and for HER2+: 71%, 24%, 14%, and 5%, respectively (Table 2).

**Table 2:** Landmark overall survival and estimated median overall survival by hormone receptor subtype

	All patients			TNBC			HR+/HER2−			HER2+		
	Eribulin (*N* = 229)	Gemcitabine (*N* = 134)	Capecitabine (*N* = 80)	Eribulin (*N* = 66)	Gemcitabine (*N* = 36)	Capecitabine (*N* = 20)	Eribulin (*N* = 142)	Gemcitabine (*N* = 77)	Capecitabine (*N* = 41)	Eribulin (*N* = 21)	Gemcitabine (*N* = 21)	Capecitabine (*N* = 19)
Landmark survival (*n*, % surviving)												
6 months	149 (65.1%)	74 (55.2%)	39 (48.8%)	32 (48.5%)	18 (50.0%)	9 (45.0%)	102 (71.8%)	39 (50.7%)	19 (46.3%)	15 (71.4%)	17 (81.0%)	11 (57.9%)
12 months	87 (38.0%)	40 (29.9%)	24 (30%)	23 (34.9%)	11 (30.6%)	5 (25.0%)	59 (41.6%)	21 (27.3%)	10 (24.4%)	5 (23.8%)	8 (38.1%)	9 (47.4%)
24 months	32 (14.0%)	16 (11.9%)	10 (12.5%)	13 (19.7%)	4 (11.1%)	3 (15.0%)	16 (11.3%)	8 (10.4%)	5 (12.2%)	3 (14.3%)	4 (19.1%)	2 (10.5%)
36 months	16 (7%)	9 (6.7%)	8 (10%)	5 (7.6%)	1 (2.8%)	3 (15.0%)	9 (7.0%)	5 (6.5%)	3 (7.3%)	1 (4.8%)	3 (14.3%)	2 (10.5%)
Estimated median OS (95%CI)												
Months (95% CI)	9.8 (8.3, 12.8)	7.2 (5.8, 10.3)	9.1 (6.3, 15.4)	7.0 (5.2, 12.9)	6.0 (3.3, 11.8)	5.5 (1.8, 9.4)	11 (8.5, 14.1)	6.4 (4.8, 8.5)	9.1 (3.3, NR)	10.3 (6.0, NR)	12.8 (7.0, NR)	15.4 (7.6, NR)

*CI* confidence interval, *HER2* human epidermal growth factor receptor 2, *HR* hormone receptor, *NR* not reached, *OS* overall survival, *TNBC* triple negative breast cancer

Landmark survival at 6, 12, 24 and 36 months for all patients treated with gemcitabine was 55%, 30%, 12%, and 7%, respectively. Corresponding landmark survival for each time point by hormone receptor subtype for patients treated with gemcitabine were as follows: for TNBC: 50%, 31%, 20%, and 3%, respectively, for HR+/HER2−: 51%, 27%, 10%, and 3%, respectively, and for HER2+: 81%, 38%, 19%, and 14%, respectively (Table 2).

Landmark survival at 6, 12, 24 and 36 months for all patients treated with capecitabine was 49%, 30%, 13%, and 10%, respectively. Corresponding landmark survival by hormone receptor subtype for each time point for patients treated with capecitabine were as follows: for TNBC: 45%, 25%, 15%, and 15%, respectively, for HR+/HER2−: 46%, 24%, 12%, and 7%, respectively; and for HER2+: 58%, 47%, 11%, and 11%, respectively (Table 2).

**Fig. 1:**
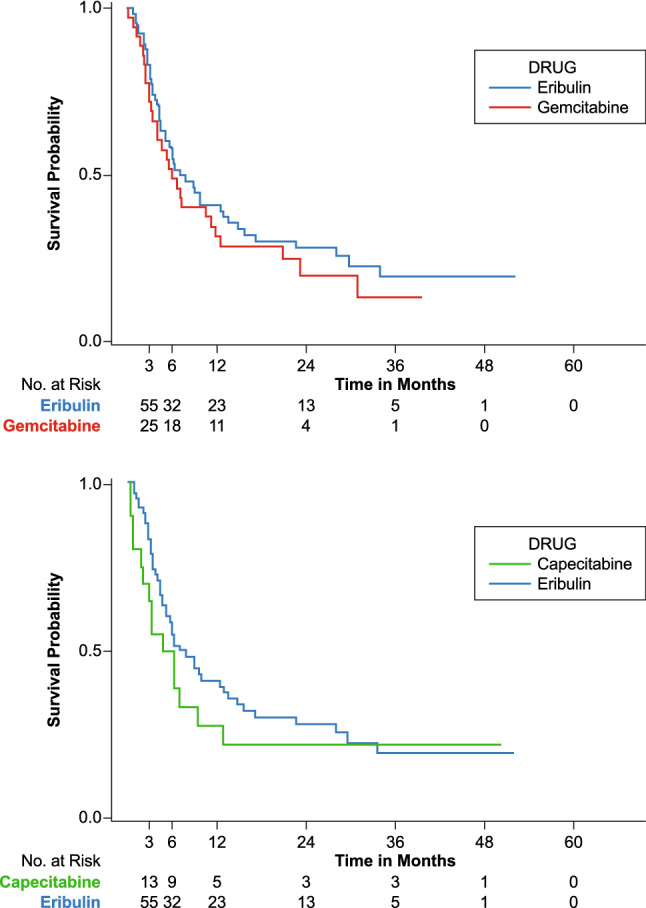
Overall survival Kaplan–Meier graph (eribulin vs gemcitabine and eribulin vs capecitabine) in metastatic triple-negative breast cancer (TNBC) patients

**Fig. 2:**
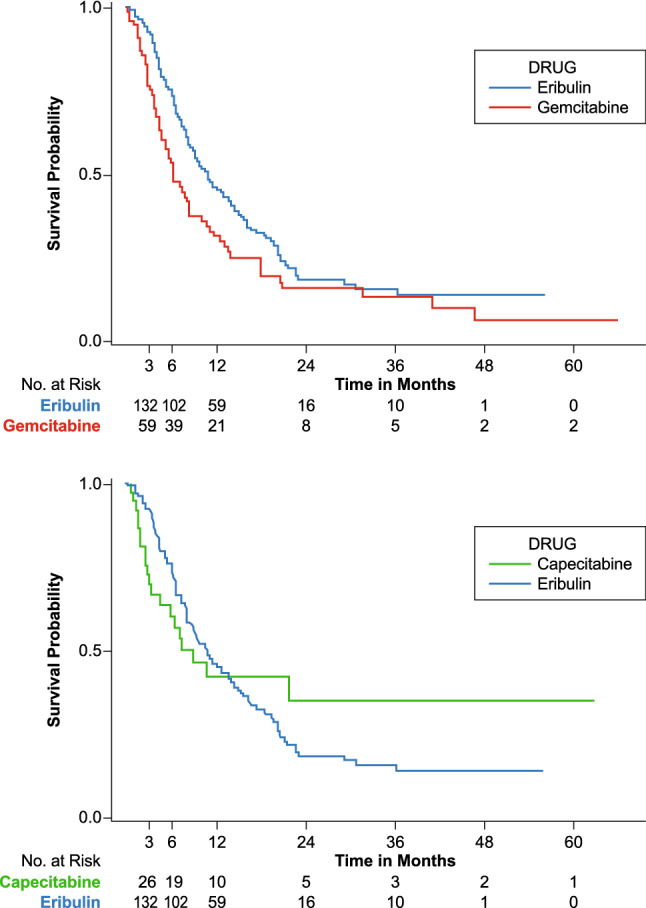
Overall survival Kaplan–Meier graph (eribulin vs gemcitabine and eribulin vs capecitabine) in metastatic HR+/HER2− patients

The median OS in the all-patient cohort was 9.8 months (95% CI 8.3, 12.8) for eribulin, 7.2 months (95% CI 5.8, 10.3) for gemcitabine, and 9.1 months (95% CI 6.3, 15.4) for capecitabine (Table 2). Median overall survival with eribulin was numerically higher in TNBC patients and HR+/HER2− patients, compared to HER2+ patients. (Table 2), though the small sample size of the HER2+ makes it challenging to make comparisons. Survival analysis for the three treatments stratified by hormone receptor subtype showed a trend favoring eribulin within the TNBC patient subtype compared to gemcitabine [HR (95% CI) 0.82 (0.51, 1.30)] and capecitabine [HR (95% CI) 0.77 (0.43, 1.38)], though it was not statistically significant. Lack of statistical significance could potentially be due to these small sample sizes (Fig. 1). Survival analysis within HR+/HER2− patient subtype showed a trend favoring eribulin compared to gemcitabine [HR (95% CI) 0.69 (0.51, 0.95)]. No statistical significance was observed between eribulin and capecitabine [HR (95% CI) 1.04 (0.64, 1.67)] (Fig. 2). No statistical differences were noted in survival for HER2+ patients between eribulin and gemcitabine or capecitabine.

## Discussion

Overall, patients with MBC and visceral metastases who received eribulin had a numerically higher percentage of patients alive at all landmark timepoints compared to gemcitabine, and a higher percentage of patients alive until the 36-month landmark compared to capecitabine. Median survival times showed that in the overall patient cohort and within the TNBC and HR+/HER2− subtype, patients receiving eribulin had a numerically higher median overall survival.

Within the TNBC subtype, patients receiving eribulin had a longer estimated median OS compared to gemcitabine or capecitabine. For the HR+/HER2− subgroup, eribulin had a higher percentage of patients alive for the first 6 and 12 months compared to gemcitabine or capecitabine. Similar to the TNBC treatment arm, the estimated median OS was also longer for HR+/HER2− patients treated with eribulin. The HER2+ subgroup showed varied results for landmark survival across treatments, and median OS was longest in the capecitabine treatment arm. In addition to the small sample size, it should be noted that capecitabine is also approved for combination therapy in the US for HER2+ patients [11] and some of the patients in capecitabine HER2+ group could have received additional therapy which may have influenced OS. Across treatments, TNBC patients had a shorter median survival than patients who were HER2+ or HR+/HER2−, which is consistent with the prognosis in the literature overall, and in numerous clinical trials.

In this study, we have noticed a modest variation in the proportion of the HR+/HER2− subtype within each of these treatment cohorts, and determined the OS results by subtype within each treatment arm. We thus feel it is unlikely that the modest difference in HR+/HER2− incidence in each of these cohorts would account for these differences in OS. In addition, cyclin-dependent kinase (CDK) 4/6 inhibitors have been approved as initial therapy for patients with HR+/HER2− MBC in the US since 2015. In this study the proportion of HR+/HER2− patients with exposure to a CDK 4/6 inhibitor before receiving 3^rd^ line chemotherapy was 21.8% in the eribulin group, 14.3% in the gemcitabine group, and 39% in the capecitabine group, respectively. However, there is no evidence to date that prior therapy with a CDK 4/6 inhibitor has an effect on subsequent OS, as determined from the initiation of latter line chemotherapy. OS in this analysis is calculated from the date of initiation of each of these three chemotherapy agents and not from the diagnosis of MBC, thus, we do not believe the difference in CDK 4/6 inhibitor use would influence our survival analyses.

In a pooled analysis of data from two phase 3 clinical trials with eribulin (Studies 301 and 305), OS was significantly longer for patients who received eribulin vs the control arm (TPC or capecitabine) (HR (95% CI) 0.85 (076, 0.94); *P* < 0.01). The median OS was 15 months for the eribulin cohort vs 12.6 months for the control arm. The pooled analysis also showed a significant difference in PFS favoring eribulin. It should be noted that the comparators in these trials were TPC or capecitabine, and were not limited to gemcitabine or capecitabine, as is the case with our analysis [13].

Eribulin has been evaluated in a real-world U.S. community oncology setting in patients with metastatic triple-negative breast cancer (mTNBC). The authors found that in a real-world setting, patients with metastatic TNBC treated with eribulin had more sites of metastatic disease and exposure to greater numbers of prior therapies than patients who were included in randomized clinical trials [15]. Our analysis included patients who were triple negative, but our analysis did not limit patients by tumor hormone status and in addition focused on patients with liver or lung metastases.

Eribulin has also been evaluated in real-world setting outside of the U.S. Patients treated with eribulin at 10 Italian hospitals from January 2012 to July 2013 were evaluated for response and survival. Similar to our analysis, this study contained patients with various tumor hormone status types, but it should be noted that the authors found that about 20% of patients evaluated did not match the EMA indication for eribulin. In Europe, eribulin is indicated for the treatment of adult patients with locally advanced or metastatic breast cancer who have progressed after at least one chemotherapeutic regimen for advanced disease. Prior therapy should have included an anthracycline and a taxane in either the adjuvant or metastatic setting unless patients were not suitable for these treatments [16]. Furthermore, the study did not focus on patients with visceral metastases. The median OS noted in the study was 11.6 months (range 0.6–33.3 months; 95% CI 8.7–14.5), which was similar to the findings of our analysis [17]. Importantly, consideration should be given to variability in treatment patterns based on the country the study was conducted in, as the label indication for treatment with eribulin varies across countries (e.g. patients in the U.S. who receive eribulin are typically heavily pretreated in line with U.S. label indication).

## Limitations

As results presented in this study are from patients from CTCA, the findings may not be generalizable to all patients with MBC and visceral metastases. Sample sizes, particularly for the capecitabine group and HER2+ subgroups were small, which may have impacted the results. As is the case with all observational, non-randomized, retrospective studies, unmeasured confounding may be present. Information on subsequent or combination therapies was not captured or controlled for in our study, which could vary by the treatment and could potentially impact outcomes, particularly overall survival. Tolerability and adverse effects were not captured.

## Conclusion

Our retrospective real-world analysis of patients with MBC and visceral metastases demonstrated that landmark overall survival was numerically higher with eribulin at 12 and 24 months compared to gemcitabine and capecitabine. Across all patients and in the TNBC and HR+/HER2− subtypes, median OS was highest in patients who received eribulin. However, survival times varied when further analyzed by hormone receptor status. In the HR+/HER2− subtype, a trend favoring eribulin versus gemcitabine was observed, and a trend favoring eribulin was noted in the TNBC subtype, though differences were not statistically significant. The study demonstrates consistency of eribulin effectiveness in MBC patients with visceral metastases across the TNBC and HR+/HER2− subtypes. Additional research is needed to better understand the impact of chemotherapy selection in patients with MBC with lung or liver visceral metastases, particularly in relation to hormone receptor status.
