# Correction to: Overall survival analysis in patients with metastatic breast cancer and liver or lung metastases treated with eribulin, gemcitabine, or capecitabine

**DOI:** 10.1007/s10549-021-06223-6

**Published:** 2021-04-27

**Authors:** Shayma Kazmi, Debanjana Chatterjee, Dheeraj Raju, Rob Hauser, Peter A. Kaufman

**Affiliations:** 1 Cancer Treatment Centers of America, Philadelphia, PA USA; 2 US Health Economics Outcomes Research and Real World Evidence, Eisai Inc., Woodcliff Lake, NJ USA; 3 Cancer Treatment Centers of America Global, Inc., Boca Raton, FL USA; 4 Larner College of Medicine, Division of Hematology/Oncology, University of Vermont Cancer Center, Burlington, VT USA

## Correction to: Breast Cancer Research and Treatment (2020) 184:559–565 10.1007/s10549-020-05867-0

In the original publication, two values provided for landmark survival for patients treated with gemcitabine were incorrectly listed in the text. On page 562, paragraph 3, the text listed that “for TNBC: 50%, 31%, 20%, and 3%, respectively, for HR+/HER2−: 51%, 27%, 10%, and 3%, respectively”. The correct values should be “for TNBC: 50%, 31%, 11%, and 3%, respectively, for HR+/HER2−: 51%, 27%, 10%, and 7%, respectively”. The correct values are consistent with those listed in Table 2.

